# The ligand-bound thyroid hormone receptor in macrophages ameliorates kidney injury via inhibition of nuclear factor-κB activities

**DOI:** 10.1038/srep43960

**Published:** 2017-03-08

**Authors:** Fumihiko Furuya, Toshihisa Ishii, Shogo Tamura, Kazuya Takahashi, Hidetoshi Kobayashi, Masashi Ichijo, Soichi Takizawa, Masahiro Kaneshige, Katsue Suzuki-Inoue, Kenichiro Kitamura

**Affiliations:** 1 Third Department of Internal Medicine, Interdisciplinary Graduate School of Medicine and Engineering, University of Yamanashi, 1110 Shimokato, Chuo, Yamanashi, 4093898, Japan; 2 Department of Laboratory and Medicine, Interdisciplinary Graduate School of Medicine and Engineering, University of Yamanashi, 1110 Shimokato, Chuo, Yamanashi, 4093898, Japan

## Abstract

In chronic kidney disease (CKD) patients, inflammation plays a pivotal role in the progression of renal fibrosis. Hypothyroidism is associated with an increased occurrence of atherosclerosis and inflammation, suggesting protective roles of thyroid hormones and their receptors against inflammatory processes. The contribution of thyroid hormone receptors to macrophage differentiation has not been well documented. Here, we focused on the endogenous thyroid hormone receptor α (TRα) in macrophages and examined the role of ligand-bound TRα in macrophage polarization-mediated anti-inflammatory effects. TRα-deficient irradiated chimeric mice showed exacerbated tubulointerstitial injury in a unilateral ureteral obstruction model. Compared with wild-type macrophages, macrophages isolated from the obstructed kidneys of mice lacking TRα displayed increased expression of proinflammatory cytokines that was accompanied by enhanced nuclear translocation of p65. Comparison of TRα-deficient bone marrow-derived macrophages with wild-type macrophages confirmed the propensity of the former cells to produce excessive IL-1β levels. Co-culture of these macrophages with renal epithelial cells induced more severe damage to the epithelial cells via the IL-1 receptor. Our findings indicate that ligand-bound TRα on macrophages plays a protective role in kidney inflammation through the inhibition of NF-κB pathways, possibly by affecting the pro- and anti-inflammatory balance that controls the development of CKD.

Thyroid hormone (T3) influences a variety of physiological processes in mammals, including cell growth and differentiation^1^. The effects of T3 are mediated by its binding to nuclear hormone receptors, thyroid hormone receptor α (TRα) and TRβ, which then associate as homodimers with another TR or as heterodimers with retinoid X receptors. These dimers act as ligand-dependent transcription factors for thyroid hormone response elements located in the regulatory regions of target genes^2^. Similar to other nuclear hormone receptors, TRs can also be activated in the cytoplasm, where they interact with and can activate kinase pathways^3^, including the AKT and MAPK phosphatase 1 (MKP1) pathways^4^. The MKP1 phosphatase can inhibit NF-κB and inflammation pathways. A recent report has indicated that ligand-bound TRα in macrophages down-regulates lipopolysaccharide-induced IL-1β expression, which is a target of the NF-κB pathway, during acute and chronic inflammation of the liver^5^. Thus, thyroid hormones and TRs may function in modulation of the inflammatory response.

Chronic kidney disease (CKD) constitutes a major public health concern. Fibrosis is a characteristic hallmark that determines the prognosis of any kidney disease and inevitably leads to kidney failure. The histological characteristics of interstitial fibrosis in the kidney are the presence of tubular atrophy and dilation, interstitial leukocyte infiltration, accumulation of fibrosis, and increased interstitial matrix deposition^6^. Among bone marrow-derived cell lineages, macrophages play a prominent role in regulating kidney fibrosis^7^, and the extent of fibrosis predicts organ failure in chronic diseases of the kidneys and other tissues^8^.

Macrophages show significant functional heterogeneity, as local environmental factors shape their properties and activation states^9^,^10^. Different stimuli activate macrophages to express distinct patterns of chemokines, surface markers, and metabolic enzymes, which ultimately generates the diversity of macrophage function observed in inflammatory and noninflammatory settings. Thus, two distinct phenotypes of polarized activated macrophages have been defined: the classically activated (M1) phenotype and the alternatively activated (M2) phenotype^9^. Macrophages are known to consist of subsets with varying cell surface markers, chemokine receptors and roles in injury and repair versus fibrosis. In bone marrow, a large population of mature monocytes are identified by the expression of CD11b^7^. Ly6C-expressing macrophages are the macrophages that typically infiltrate tissue and are implicated in disease processes and these macrophages have been termed inflammatory or M1 macrophages. Previous reports indicate that CD11b^+^ Ly6C^high^ macrophages promote kidney damage and fibrosis^11^. In contrast, Ly6C^low^ macrophages exhibit low expression of pro-inflammatory cytokines. Classically activated macrophages are induced by proinflammatory mediators such as LPS and IFN-γ. These activated macrophages exhibit enhanced proinflammatory cytokine production (TNFα, IL-6, or IL-12) and generate reactive oxygen species such as NO, via activation of iNOS. The heterogeneity of macrophage polarization plays a critical role in the pathogenesis of kidney damage and fibrosis^12^. Activation of these cells towards a proinflammatory state determines the local composition of cytokine reservoirs that orchestrate disruptions in the renal architecture following an insult.

Aberrant activation of NF-κB is associated with metabolic disease and CKD^13^. The NF-κB pathway unites inflammatory and metabolic responses and, as a well-studied mediator of inflammation and immunity, this pathway represents an important target for better understanding metabolic diseases and developing novel therapeutic strategies. The inflammatory response is characterized by the coordinated activation of various signaling pathways that regulate the expression of proinflammatory mediators in resident tissue cells and in monocytes recruited from the blood. In the current study, we used TRα-deficient macrophages to determine whether ligand-bound TRα depletion in macrophages exacerbates CKD by promoting alternative M1 macrophages in the kidneys. We employed the unilateral ureteral obstruction (UUO)-induced kidney injury model for CKD because UUO stimulates the renin-angiotensin system, and macrophages are critical to the pathogenesis of kidney fibrosis^14^. We found that TRα on macrophages protects the kidneys and acts as an endogenous inhibitor of kidney inflammation.

## Results

**Figure 1:**
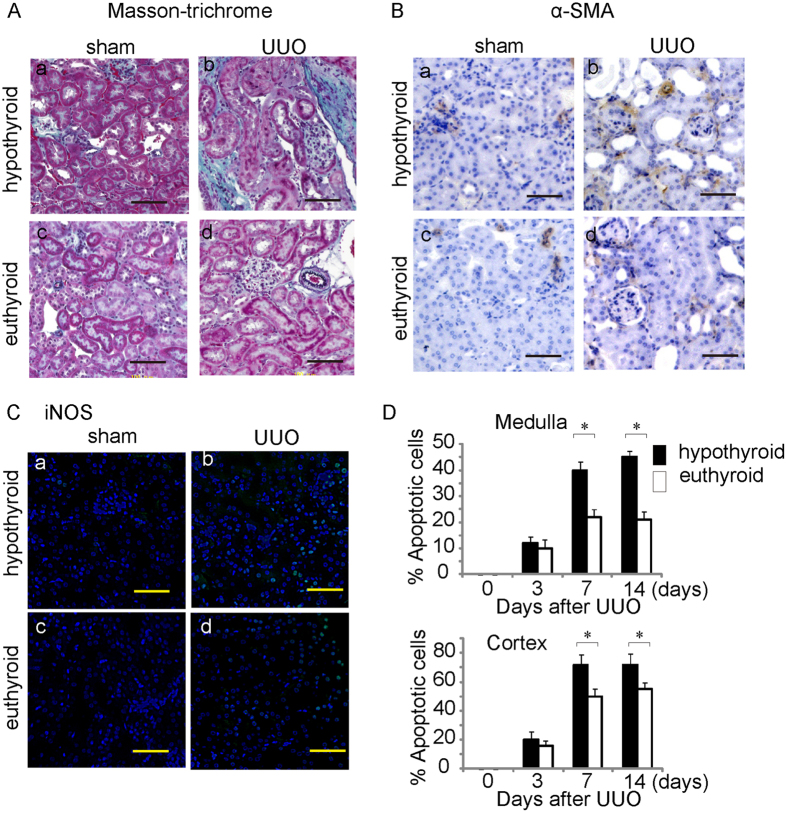
Hypothyroid conditions exacerbate UUO-induced kidney injury. (**A**) Masson’s trichrome staining of hypothyroid or euthyroid mouse kidneys 14 days after Sham treatment or UUO. Scale bars represent 50 μm. (**B**) Immunostaining to detect UUO-induced expression of α-smooth muscle actin in the kidneys of hypothyroid or euthyroid mice 14 days after Sham treatment or UUO. Scale bars represent 50 μm. (**C**) Immunostaining to detect UUO-induced expression of iNOS in the kidneys of hypothyroid or euthyroid mice 14 days after Sham treatment or UUO. Nuclei were stained with DAPI. Scale bars represent 50 μm. (**D**) Percentages of TUNEL-positive epithelial cells among the cells with DAPI-stained nuclei in the medulla or cortex. *p < 0.001. n = 6.

The thyroid hormone triiodothyronine T3 influences a variety of physiological processes in mammals, including cell growth and metabolism. First, we analyzed the effects of normal or hypothyroid conditions on the kidney. Hypothyroid mice were provided a low-iodine diet and methimazole in their drinking water. Neither group of mice exhibited kidney dysfunction (data not shown). However, when the hypothyroid mice were subjected to UUO, they displayed severe structural destruction of the kidneys compared with euthyroid mice with UUO, as indicated by deterioration of tubular dilation and atrophy (Fig. 1A). In the kidneys of the hypothyroid mice, UUO increased the accumulation of α-SMA, which is a marker of myofibroblasts, mesangial cells, or smooth muscle cells (Fig. 1B). Histological findings indicated that the number of macrophages expressing iNOS as a proinflammatory macrophage marker was significantly increased in the obstructed kidneys of hypothyroid mice compared with euthyroid mice (Fig. 1C). Moreover, TUNEL staining indicated that a significantly greater number of apoptotic cells were present in both the cortex and medulla of the kidneys of hypothyroid mice compared with those of euthyroid mice (Fig. 1D). Thus, interstitial fibrosis was significantly exacerbated in hypothyroid mice compared with euthyroid mice. These results indicated that the T3 signal might ameliorate kidney injury in the UUO model of tubulointerstitial disease.

**Figure 2:**
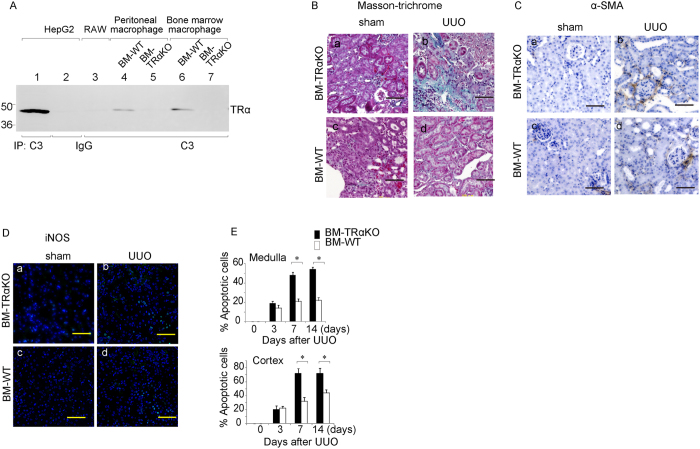
The kidneys of BM-TRα KO mice are more susceptible to UUO. (**A**) Western blot of TRα expression in macrophages from BM-WT or BM-TRα KO mouse kidneys. Cell lysates were first immunoprecipitated with either a TRα antibody (C3) that recognizes the TRα C terminus or normal mouse IgG. Western blot analysis of the precipitates was then performed using an antibody against the TRα N-terminus. The same amount of protein extract prepared from HepG2 cells was used as a positive or negative control. The RAW macrophage cell line was also analyzed. (**B**) Masson’s trichrome staining of BM-WT or BM-TRα KO mouse kidneys 14 days after Sham treatment or UUO. Scale bars represent 50 μm. (**C**) Immunostaining to detect UUO-induced expression of α-smooth muscle actin in the kidney of BM-WT or BM-TRα KO mice 14 days after Sham treatment or UUO. Scale bars represent 50 μm. (**D**). (**C**). Immunostaining to detect UUO-induced expression of iNOS in the kidney of BM-WT or BM-TRα KO mice 14 days after Sham treatment or UUO. Nuclei were stained with DAPI. Scale bars represent 50 μm. (**E**) The percentage of TUNEL-positive epithelial cells among the cells with the DAPI-stained nuclei in the medulla or cortex. *p < 0.001. n = 6.

Recent studies have suggested that inflammation is crucially involved in kidney cellular injury and fibrosis^15^. Indeed, Fig. 1C shows that UUO induced the accumulation of iNOS-positive macrophages in hypothyroid mice. The actions of T3 are mediated by binding to the nuclear hormone receptor TR. To analyze the function of ligand-bound TRα of monocytes/macrophages in the UUO-kidney, C57BL/6 male mice were subjected to intravenous bone marrow transplantation from TRα-deficient^16^ or wild-type mice to generate TRα-deficient irradiated chimeric mice (BM-TRα KO) or control mice (BM-WT), respectively. These reconstituted mice were used for UUO experiments 7 weeks after irradiation and bone marrow transplantation. In the kidneys or peritoneal cavity, F4/80-expressing macrophages were labeled and isolated using fluorescent sorting strategies. As shown in the Western blot analysis presented in Fig. 2A, TRα was clearly expressed in macrophages in the kidney, including in both the peritoneal cavity macrophages and bone marrow-derived macrophages of BM-WT mice. In contrast, TRα expression was not observed in the macrophages of BM-TRα KO mice. Endogenous TRα expression was not observed in RAW 264.7 cells, a macrophage cell line. To evaluate the actions of TRα in macrophages that infiltrate kidneys undergoing fibrosis, we harvested kidneys from BM-TRα KO or BM-WT mice with UUO. The obstructed kidneys from BM-TRα KO mice exhibited a higher level of interstitial fibrosis and more iNOS-positive and apoptotic cells than BM-WT mice (Fig. 2B–E). These results indicated that TRα deficiency in macrophages critically exacerbated tubular epithelial cell damage and fibrosis.

**Figure 3:**
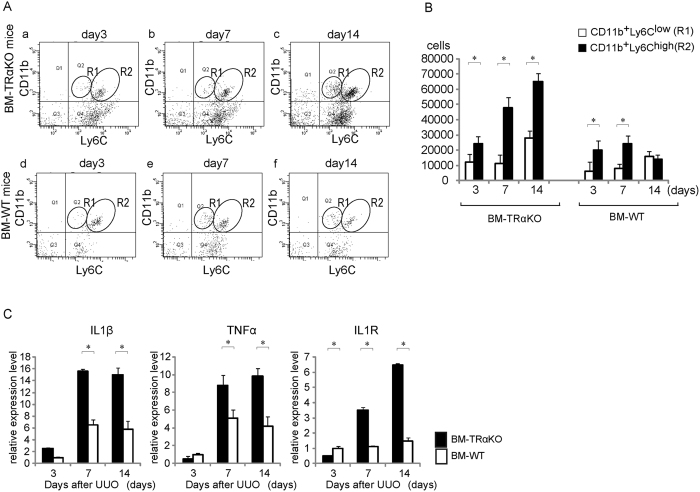
Ligand-bound TRα controls the recruitment and accumulation of CD11b^+^ Ly6C^+^ cells in response to UUO. (**A**) Representative flow cytometric plots of macrophages in the whole kidneys of BM-TRα KO (a–c) or BM-WT (d–f) mice that underwent the UUO operation. R1 and R2 indicate CD11b^+^ Ly6C^low^ and CD11b^+^ Ly6C^high^ cells, respectively. (**B**) Fractions of CD11b^+^ Ly6C^low^ (R1) and CD11b^+^ Ly6C^high^ (R2), which are subpopulations of CD11b^+^ Ly6C^+^ cells (Q2) isolated from the kidneys of BM-TRα KO or BM-WT mice subjected to UUO. All data are expressed as the means ± S.D. (error bars). *p < 0.001. n = 6. (**C**) The levels of IL-1β, TNFα, and IL1R mRNA were determined by quantitative real-time RT-PCR using 50 ng of cDNA, in triplicate. Relative quantification of target cDNA was performed by arbitrarily setting the control value from BM-WT in obstructed kidneys on day 3 after UUO to 1. All data are expressed as the means ± S.D. (error bars). *p < 0.05 compared with BM-TRα KO mice. n = 6.

CD11b^+^ Ly6C^+^ macrophages are recruited from blood, and CD11b^+^ Ly6C^high^ macrophages promote tissue damage and fibrosis^7^,^17^. On days 3, 7, and 14 after UUO, UUO induced the accumulation of CD11b^+^ Ly6C^+^ (Q2) cells, which consisted of 2 major subpopulations: CD11b^+^ Ly6C^low^ (R1) and CD11b^+^ Ly6C^high^ (R2) cells, in BM-WT and BM-TRα KO mice (Fig. 3A). On days 7 and 14 after UUO, the accumulation of CD11b^+^ Ly6C^low^ macrophages in the kidneys was increased in BM-WT mice; in contrast, CD11b^+^ Ly6C^high^ macrophages were still predominant in the BM-TRα KO mice (Fig. 3B). The ratio of R2/R1 on day 7 in BM-TRαKO mice was significantly higher than that in BM-WT mice (Supplementary Figure 1A). The knock-down of TRα significantly enhanced macrophage migration in Boyden chambers (Supplementary Figure 1B).

Next, to examine the effects of TRα on the polarization of bone marrow-derived macrophages, CD11b^+^ Ly6C^+^ macrophages were directly isolated from the obstructed kidney using a cell sorter. The infiltrating macrophages of the obstructed kidneys from BM-TRα KO mice showed significantly higher expression of proinflammatory markers, including IL-1β, TNFα, and IL1R, than BM-WT kidneys at 7 and 14 days after UUO (Fig. 3C). These results suggest that CD11b^+^ Ly6C^high^ and CD11b^+^ Ly6C^low^ cells differentially accumulate in the kidney during the course of the response to UUO. At early stages of UUO, during tissue destruction, it was primarily CD11b^+^ Ly6C^high^ macrophages that accumulated in the kidneys. Later, after 7 days of UUO, when tissue remodeling and fibrosis were dominant, the numbers of CD11b^+^ Ly6C^low^ cells were increased in BM-WT mice. TRα deficiency in bone marrow enhanced the accumulation of CD11b^+^ Ly6C^high^ cells and altered the balance of macrophage polarity during the response to UUO. These results indicated that ligand-bound TRα constrains infiltrating macrophage activation by suppressing key inflammatory cytokines, including those involved in the IL-1β pathway, during kidney injury.

**Figure 4:**
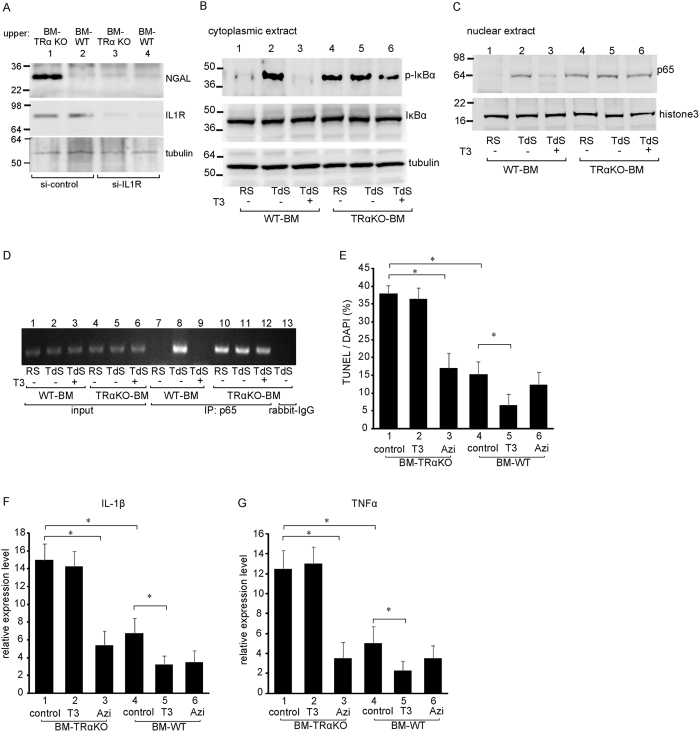
TRα-deficient macrophages induce tubular epithelial cell injury. (**A**) Murine tubular epithelial cell lines (M1 cells) were cocultured with CD11b^+^ Ly6C^+^ macrophages that were isolated from BM-WT or BM-TRα KO mice and passaged on the upper layer of Transwell plates. The effect of siRNA knockdown of the IL1R (or control siRNA) on the expression level of NGAL and the IL1R in CD11b^+^ Ly6C^+^ cells from BM-WT or BM-TRα KO mice was analyzed using Western blotting. Equal protein loading was evaluated by assessing tubulin levels. (**B**,**C**) WT-BM or TRα KO-BM were pooled from 6 mice and incubated for 24 hours in 10% serum (RS), stripped serum (TdS), or TdS with 30 nM of T3. The expression of phosphorylated-IκBα (p-IκBα) and total IκBα in the cytoplasmic fraction (**B**) and of p65 in the nuclear fraction (**C**) was analyzed. The expression of tubulin or histone 3 was used as a loading control for the cytosolic and nuclear fractions, respectively. (**D**) ChIP assay using TRαKO-BM or WT-BM cells. Anti-p65 antibodies or normal rabbit IgG were used for immunoprecipitation (IP) of the chromatin. Input indicates 10% of the chromatin. (**E**) Percentages of TUNEL-positive epithelial cells among the cells with DAPI-stained nuclei in the kidneys of BM-TRαKO or BM-WT mice. T3, T3-treatment group; Azi, Azithromycin-treatment group. All data are expressed as the means ± S.D. (error bars). *p < 0.05. The levels of IL-1β (**F**) or TNFα (**G**) mRNA were determined by quantitative real-time RT-PCR. Relative quantification of target cDNA was performed by arbitrarily setting the control value of BM-WT in obstructed kidneys on day 3 after UUO to 1 (Fig. 3).

To clarify the effects of inappropriate macrophage polarity due to TRα deficiency on tubular epithelial cells, we analyzed the expression of NGAL, a marker of kidney injury, in kidney epithelial cell lines following co-culture with CD11b^+^ Ly6C^+^ macrophages from the kidneys of BM-TRα KO or BM-WT mice on day 7 after UUO. NGAL protein expression was strongly enhanced in epithelial cells that were incubated with TRα-deficient macrophages isolated from the kidneys at 7 days after UUO compared with cells that were incubated with BM-WT macrophages (Fig. 4A, lane 1 vs. 2). These results indicated that cytokines from activated macrophages that lack TRα could induce a fatal disorder of kidney epithelial cells, without direct contact with the macrophages. Furthermore, we analyzed whether TRα-deficient macrophages exacerbated tubular cell damage through functional activation of IL1R. IL1R-deficient tubular cells were co-cultured with macrophages lacking TRα. NGAL expression was completely inhibited in IL1R KO renal tubular cells that were co-incubated with macrophages from BM-WT mice. The NGAL expression level induced by TRα KO macrophages was lower than that in cells transfected with control-siRNA (Fig. 4A, lane 1 vs. 3. These results indicated that activation of the IL1R in kidney epithelial cells in response to IL-1β secreted from activated macrophages triggers kidney cell injury.

To explore the molecular mechanisms by which TRα-deficient macrophages induced the expression of proinflammatory cytokines, we focused on NF-κB, which is an important transcription factor involved in regulating inflammatory processes. Bone marrow-derived macrophages were cultured in one of three serum conditions: regular serum, T3-depleted serum, or 30 nM T3-containing serum for 48 hours. Subsequently, the cytoplasmic and nuclear kinetics of NF-κB were analyzed using Western blotting (Fig. 4B and C). As shown in Fig. 4B, phosphorylated IκBα (p-IκBα) was not observed in WT macrophages (lane 1). The abundance of p-IκBα was increased after T3 depletion (lane 2), whereas supplementation with T3 abrogated the phosphorylation of IκBα (lane 3) in the bone marrow-derived macrophages of WT mice (WT-BM) but not in the bone marrow-derived macrophages of TRαKO mice (TRαKO-BM) (lanes 4–6). The total IκBα protein levels in the cytoplasmic extracts of WT-BM and TRαKO-BM cells were not altered by the T3 treatment. As shown in Fig. 4C, little nuclear p65 was observed in WT-BM that was cultured with regular serum (lane 1). However, nuclear p65 levels were significantly increased after T3 depletion (lane 2), and T3 treatment significantly suppressed nuclear p65 levels (lane 3) in WT-BM. In contrast, nuclear p65 levels remained high and did not differ significantly in TRαKO-BM cells under all conditions (lanes 4–6). Tubulin and histone 3 levels were used as controls to validate the integrity of the cytoplasmic and nuclear extracts, respectively. Detection of the nuclear marker histone3 only in the nuclear fraction and not in the cytosolic fraction, and detection of the cytoplasmic marker tubulin only in the cytosolic fraction and not in the nuclear fraction is shown in Supplementary Figure 2.

To determine if TRαKO-BM production of IL-1β is governed by p65, we performed chromatin immunoprecipitation (ChIP) assays on WT-BM or TRαKO-BM cells with or without T3 treatment. No interaction of p65 with the IL-1β promoter was detected in WT-BM cells that were cultured in regular serum. p65 was associated with fragments of the IL-1β promoter when these cells were incubated in T3-stripped serum but not when they were incubated with T3 (Fig. 4D). In contrast, p65 interaction with the IL-1β promoter DNA was observed in TRαKO-BM cells with or without T3-treatment.

Next, to analyze the effects of a hyperthyroid condition on kidney injury, BM-WT or BM-TRαKO mice were treated with T3 every day for 7 days after UUO. TUNEL staining indicated that there was no difference in the number of apoptotic cells between with or without T3-treatment in BM-TRαKO mice. In contrast, significantly fewer apoptotic cells were observed in the kidneys from T3-treated BM-WT mice, compared with T3-untreated mice (Fig. 4E). These results indicated that T3 had protective functions against kidney injury and that a deficiency of ligand-bound TRα in macrophages critically exacerbated kidney injury. The above findings also predicted that inhibition of the NF-κB-pathway would improve the kidney injury in BM-TRαKO mice with UUO. To test this hypothesis, we treated BM-TRαKO mice with Azithromycin, a poten NF-κB inhibitor^18^, and evaluated the effects of Azithromycin on UUO-induced kidney injury. TUNEL staining indicated that Azithromycin-treatment decreased the number of apoptotic cells in the kidneys of BM-TRαKO mice with UUO (Fig. 4E). Azithromycin-treatment did not inhibit the apoptosis in BM-WT mice with UUO.

The mRNA expression of IL-1β or TNFα was not significantly altered between with or without T3-treatment in CD11b^+^ Ly6C^+^ macrophages of BM-TRαKO mice with UUO (Fig. 4F or G, respectively). However, Azithromycin-treatment significantly decreased the expression of both IL-1β and TNFα mRNA in CD11b^+^ Ly6C^+^ macrophages of BM-TRαKO mice with UUO. T3-treatment decreased the mRNA expression of both IL-1β and TNFα in BM-WT mice. When treated with Azithromycin, the expression levels of IL-1β or TNFα were not altered in BM-WT mice with UUO. These results indicated that ligand-bound TRα inhibits activation of the NF-κB pathway by inhibiting the phosphorylation of IκBα and the translocation of p65 to the nucleus and further showed that TRα deficiency induces inappropriate activation of the NF-κB pathway.

**Figure 5:**
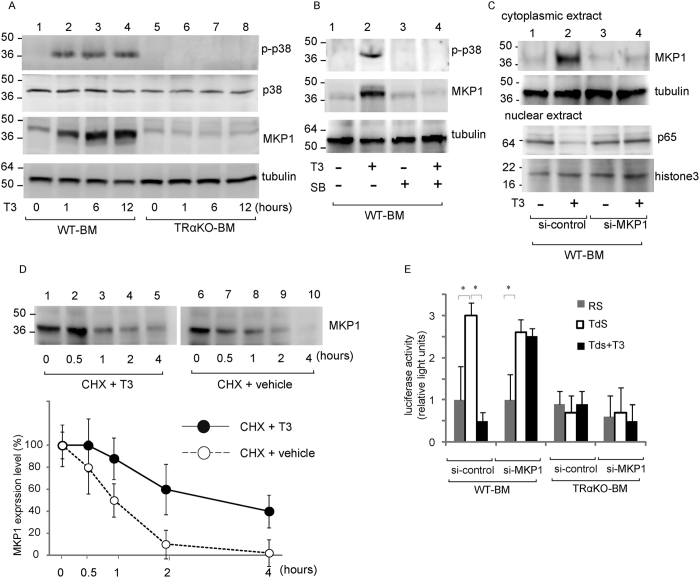
T3 induces the expression of the MKP1 protein at the posttranslational level via p38 activation, and inhibits the NF-κB pathway. (**A**) Western blot analysis of the expression levels of phosphorylated p38, total p38, and the MKP1 protein in WT-BM or TRαKO-BM cells exposed to 30 nM T3 for 1, 6, or 12 hours. (**B**) The effect of SB203580 (SB) treatment on T3-induced MKP1 expression was analyzed. (**C**) The effects of siRNA knockdown of MKP1 (or control siRNA) on the expression of MKP1 in the cytoplasmic fraction and the expression of p65 in the nucleus in WT-BM cells were analyzed. WT-BM cells were pooled from 6 mice and separated into nuclear or cytosolic fractions. The expression of tubulin or histone 3 was used as a loading control for the cytosolic and nuclear fractions, respectively. (**D**) The effect of cycloheximide (CHX) (10 μg/ml) treatment on T3-induced MKP1 expression was analyzed. (**E**) Effect of control siRNA or MKP1 siRNAs on luciferase activity in bone marrow-derived macrophages that were cotransfected with an NF-κB-driven Luc reporter and incubated for 24 hours in 10% serum (RS), stripped serum (TdS), or TdS with 30 nM of T3. Luciferase activity was normalized to Renilla activity and expressed as induction over the controls. Relative signals were determined by arbitrarily setting the value for control cultures of WT-BM and TRαKO-BM cells in the RS condition to 1. The data represent the means ± S.D. (error bars). *p < 0.05.

In pituitary cell lines, MAPK phosphatase 1 (MKP1) inactivates MAPK and inhibits the nuclear translocation of NF-κB by inducing MAPK dephosphorylation^4^,^19^. To analyze the mechanisms by which ligand-bound TRα activates the MAPK pathway and modulates the expression of MKP1 in macrophages, we used Western blot analysis to evaluate the activation of MAPK/p38 in WT-BM or TRαKO-BM precultured with T3-depleted medium (Fig. 5A). Phosphorylated-p38 (p-p38) expression was not observed in the WT-BM or TRαKO-BM mice (lanes 1 and 5) at time 0. After 1 to 12 hours of incubation with 30 nM T3, p-p38 expression was observed in WT-BM (lanes 2–4) but not in T3-treated TRαKO-BM (lanes 6–8) cells. The total p38 protein levels in WT-BM and TRαKO-BM cells were not modified by T3 treatment. MKP1 protein expression was gradually increased in WT-BM after incubation with T3 for 1, 6, or 12 hours (lanes 2–4), and MKP1 protein levels were significantly lower in TRαKO-BM than in WT-BM.

When these macrophages were exposed to T3 in combination with a specific p38 inhibitor, SB203580, for 12 hours, the ligand-bound TRα-induced expression of MKP1 was completely abolished. This change was accompanied by inhibition of p-p38 (Fig. 5B). Next, we analyzed the effect of MKP1 downregulation using MKP1-specific siRNA. WT-BM cells were transfected with either the control or MKP1 siRNA and were then incubated for 48 hours. The cells were subsequently treated with or without T3 for an additional 12 hours. The T3-induced expression of MKP1 was completely inhibited in MKP1 siRNA-transfected cells (Fig. 5C). T3 treatment diminished p65 nuclear translocation in the control cells, whereas it did not affect the level of p65 in the nucleus of macrophages that were transfected with MKP1 siRNAs. WT-BM cells were then precultured with 30 nM T3 for 12 hours, after which cycloheximide was added to the cells, with or without T3. MKP1 protein levels were measured using Western blot analysis at several subsequent time points (Fig. 5D). Incubation with T3 significantly increased MKP1 protein levels. In the presence of cycloheximide, MKP1 protein levels decayed at a lower rate in the T3-treated cells (half-life of 1 hour) than in the vehicle-treated cells (half-life of 20 minutes). These results indicated that ligand-bound TRα induced the expression of MKP1 protein at the posttranslational level through a mechanism that depended on p38 activity.

To assess the role of MKP1 in the T3-mediated inhibition of NF-κB transcription activity, macrophages were cotransfected with a NF-kB-Luc reporter together with control siRNA or MKP1 siRNAs. T3 treatment reduced NF-κB-mediated transcription to 30–40% that of control cells, and this T3-induced reduction was almost completely abolished in MKP1 siRNA-transfected cells (Fig. 5E). In contrast, MKP1 expression inhibited the activities of NF-kB in TRα-deficient macrophages. These combined findings demonstrated that the ligand-bound TRα-mediated inhibition of NF-κB pathways is mediated by p38/MAPK-induced MKP1 expression.

## Discussion

In this report, we showed, for the first time, that ligand-bound TRα can repress the activation of NF-κB involved in polarizing macrophages. We also demonstrated that ligand-bound TRα inhibited nuclear localization of p65 in macrophages through a mechanism that was dependent on MAPK and MKP1. Indeed, we showed that ligand-bound TRα enhanced the stabilization of MKP1 protein expression via MAPK, leading to down-regulation of NF-κB activity and expression of proinflammatory cytokines. TRα-deficient irradiated chimeric mice exhibited exacerbated kidney injury in the unilateral ureteral obstructed kidney. Macrophages isolated from the obstructed kidneys of mice solely lacking TRα in the bone marrow displayed increased expression of proinflammatory cytokines, including IL-1β, compared with wild-type mice. Our findings indicated that ligand-bound TRα on macrophages protects the kidney via inhibiting the NF-κB pathway, which may occur by affecting the pro- and anti-inflammatory balance that controls the development of chronic kidney disease.

The kidney is an organ that is frequently afflicted with fibrosis. Many different inflammatory triggers converge on a similar pattern of chronic inflammation of fibrosis targeting the kidney interstitium. A key role for inflammatory macrophages and their circulating progenitors (monocytes) in this process has been noted in many different organ settings^20^,^21^. However, the mechanisms by which macrophages cause fibrosis in diseases where there is continuous or repetitive injury have been controversial. Reports have implicated a subpopulation of bone marrow-derived cells known as fibrocytes that directly lay down a fibrous matrix, although the fibrocyte appears to be a minor contributing cell type in the kidneys and liver^22^,^23^. Recent studies demonstrating the diversity of macrophage phenotypes and functionality suggest that the activation state of macrophages may determine their pathogenic or reparative roles in kidney disease^24^. *In vitro* studies have shown that Th1 cytokines (TNFα and IL-1β), either alone or in concert with microbial products, elicit classical M1 activation of macrophages.

Although nuclear translocation of NF-κB has been considered the principal method for activating NF-κB-dependent gene expression, alternate mechanisms of NF-κB activation involve phosphorylation of the p65 subunit^25^. Several pathways of p65 protein phosphorylation have been identified, including phosphorylation by the IKK complex or by MAPK. This phosphorylation enhances the transactivation potential of p65. Our data also show that ligand-bound TRα decreases the nuclear translocation of p65 and its transactivation potential, which correlates with the observed decrease in NF-κB-dependent transcription. It has been previously shown that p65 and TRα do not physically interact in yeast two-hybrid systems^26^, suggesting that the repressive effect of T3 on p65 transactivation does not arise from a direct interaction between these transcription factors. However, we cannot exclude a possible interaction in the context of native promoters. An alternative mechanism for the transrepression of NF-κB activity by T3 could involve sequestration of common cofactors that are shared between NF-κB and the TR. In this context, our study provides the first evidence that ligand-bound TRα inhibition of nuclear p65 depends on MKP1 expression. It was reported that the absence of TRα activates AKT/NF-κB pathways and enhances the expression of inflammatory cytokines^27^. Our current results indicate that endogenous TRα inhibits the phosphorylation of IκBα and the nuclear-translocation of p65 that is accompanied by its association with the IL-1β promoter. We showed that impairment of MKP1 expression abrogated the effects of ligand-bound TRα and that re-expression of MKP1 rescued the termination of NF-κB activities. MKP1 is one of the components of the IKK signalosome. MKP1 is associated with IKKα, and it has been speculated that MKP1 might be the phosphatase responsible for down-regulating IKK activity^28^.

Macrophages play an essential role in communicating inflammation between metabolic tissues in response to several types of stress. In macrophages, proinflammatory pathways regulated by NF-kB contribute to vascular disease associated with metabolic excess. NF-kB integrates multiple processes in the formation of atherosclerotic plaques. NF-kB controls the expression of genes that direct the initiation and progression of atherosclerosis or fibrosis, including cytokines, such as TNFα or IL-1β. NF-κB has been detected in the nuclei of macrophages in atherosclerotic lesions^29^. In the UUO-kidney of TRα-deficient irradiated chimeric mice, infiltrating macrophages that had lost ligand-bound TRα expressed Ly6C^high^ and included a proinflammatory population. These macrophages also expressed CD11b, suggesting that they originated in the bone marrow. The *in vitro* data shown in Fig. 4 indicated that nuclear localization of p65 was observed in TRα-deficient macrophages, which was consistent with prolonged detection of CD11b + Ly6C^high^ macrophages after 14 days of UUO. These results indicated that inappropriate nuclear localization of p65 in TRα-deficient macrophages enhanced the expression of proinflammatory cytokines and that these macrophages accumulated in the UUO-kidney following severe injury.

Deiodinase type 2 (Dio2) is a thyroid hormone-activating enzyme that converts the pro-hormone T4 into the active hormone T3. Inflammation induces the expression of Dio2 in the macrophages in mouse liver^5^. It was also reported that locally produced T3 acting via the TRα has a role in the inflammatory response. In lung injury mouse models, Dio2 knockdown or hypothyroid mice are more susceptible to lung injury and demonstrate severe lung damage, which can be partly reversed by T3 administration^30^,^31^. The Dio2 Thr92Ala-allele is associated with susceptibility to osteoarthritis^32^ and sepsis^31^. The potential role of local thyroid hormone metabolism in inflammatory cells in macrophage mediated diseases such as atherosclerosis, osteoarthritis, and metabolic disease should be explored.

Several studies have indicated that TRα expression is much higher in vascular smooth muscle cells and bone marrow-derived cells than in cells from other organs, and that TRα activity can impact the level of atherosclerosis risk factors and atherosclerosis development^27^. Recent reports have indicated that T3 signaling regulates the development of unpolarized macrophages via TRβ in macrophages^33^. In the present study, T3 depletion enhanced the abundance of p-IκBα in the cytoplasm of macrophages and was associated with nuclear translocation of p65. T3 treatment decreased the phosphorylation of IκBα. Induction of MKP1 in several types of cells by other nuclear receptors, estrogen receptors or glucocorticoid receptors has been previously reported. This is the first time that ligand-bound TRα has been shown to induce MKP1 expression in macrophages. It is also significant that the ligand-bound TRα-mediated induction of MKP1 depends on the MAPK pathway. Although different mechanisms are involved in MKP1 expression, the main regulator appears to be p38/MAPK, which induces, phosphorylates, and stabilizes MKP1 by impeding its proteolytic degradation^34^. Our results demonstrate that ligand-bound TRα induces the expression of the MKP1 protein via a posttranslational mechanism, in which it decreases its degradation via the ubiquitin-proteosome pathway. *In vitro* experiments suggest that ligand-bound TRα induces MKP1 phosphorylation via a MAPK pathway.

The TRs are a superfamily of nuclear hormone receptors that include T3-dependent transcription factors. Similar to other steroid hormone receptors, several functions of TRs beyond their function as transcription factors have been reported^35^,^36^. Functional cross-talk between nuclear hormone receptors and NF-κB has been identified for various classes of receptors and has been shown to be crucial for regulating many cellular functions^37^. Peroxisome proliferator-activated receptor γ (PPARγ) reduces proinflammatory cytokine expression by antagonizing the activity of MAPK pathways and interfering with NF-κB^38^. Estradiol blocks the hypoxia-mediated activation of MAPK and NF-κB transcription^39^. Thus, nuclear hormone receptors can inhibit the action of NF-κB in the presence of their cognate ligands through a variety of mechanisms. We show here that ligand-bound TRα inhibits NF-κB-dependent transcription in macrophages by affecting the phosphorylation of IκBα, translocation of active NF-κB complexes from the cytosol to the nucleus and transactivation of p65.

The incidence of end-stage kidney disease is increasing worldwide and represents a major public health concern. Kidney interstitial fibrosis is one of the major pathological features of chronic kidney disease. After infiltrating the kidneys, macrophages can promote inflammatory injury or direct tissue repair. The kidney exhibits a remarkable ability to regenerate following acute injury. Most notably, the kidney epithelia have the intrinsic capacity to rapidly self-duplicate^40^. Kidney injury and repair involve a delicate balance between cell death and proliferation as well as macrophage–dependent interstitial matrix accumulation and remodeling. In the current study, we demonstrated that ligand-bound TRα can antagonize the IL-1β signaling pathway in proinflammatory macrophages through a mechanism that involves MKP1 induction. Moreover, these findings that show that ligand-bound TRα regulates the effects of IL-1β on both MAPK and NF-κB activation in macrophages, offer new perspectives for clarifying the function of ligand-bound TR as an anti-inflammatory ligand that controls the effects of IL-1β in macrophages.

## Methods

### Cells, cell transfection and endogenous TRα analysis

M1 cell lines (mouse tubular epithelial cell lines), the RAW246.1 cell line, and mouse macrophages were purchased from the ATCC (Manassas, VA) and maintained in DMEM containing 10% fetal bovine serum (FBS). BM was isolated from humanely culled C57BL/6-background wild-type mice (WT) or TRα-deficient mice (TRαKO), and BM-derived macrophages were obtained through *in vitro* differentiation of these samples with 100 ng/ml M-CSF in RPMI 1640 containing 10% heat-inactivated FBS for 7 days. Plasmids or siRNAs were transfected using the Lipofectamine 3000 (Thermo Fisher, Waltham, MA) or jetPEI^®^-Macrophage (Polyplus, NY) reagent according to the manufacturer’s instructions. The pGL4-luc-NFκB plasmid was purchased from Promega (Madison, WI). WT-BM and TRαKO-BM cells were transfected with 50 nM of si-control or si-MKP1, and, 24 h later were transfected with the pGl-luc-NF-κB plasmid and the Renilla luciferase plasmid, using the jetPEI-macrophage transfection reagent (Polyplus, NY) according to the manufacturer’s instructions. Transient co-transfections were carried out in triplicate. Twenty-four hours after transfection, the cells were treated with or without T3 for an additional 24 h. Dual luciferase assays were carried out according to the manufacturer’s instructions (Promega). The transfection efficiency was approximately 10–20% of the total cells (data not shown).

To analyze the expression of endogenous TRα in F4/80-expressing macrophages, the whole-cell lysate was immunoprecipitated with either a mouse monoclonal antibody that recognizes the TRα C-terminal region (C3 antibody, Santa Cruz) or a control IgG, and the immunoprecipitate was analyzed by Western blotting with an antibody against an N-terminal TRα peptide (T17 antibody, Santa Cruz)^41^.

### ChIP assay

ChIP assays were performed using a simple ChIP enzymatic chromatin immunoprecipitation kit (Cell Signaling Technology). TRαKO-BM or WT-BM cells (5 × 10^7^ cells) were fixed with 1% formaldehyde and digested chromatin was immunoprecipitated with normal rabbit IgG (negative control) or with anti-p65 antibodies (Santa Cruz). Diluted chromatin (500 μl) was immunoprecipitated with 5 μg of each antibody and protein G magnetic beads according to the manufacturer’s instructions. Quantitative real-time (RT)-PCR was performed using a SYBR green real-time PCR kit (Life Technologies). The primer sequences used for RT-PCR were: forward 5′-TCTATTTCCCTTCAGTGCTG-3′ and reverse 5′-TTCATGAGCACAGTCCATCT-3′), which correspond to the nucleotides flanking the p65 binding site in the IL-1β promoter^42^.

### Animals

The animal experimental protocol was conducted in accordance with the guidelines of the “Animal experiment rules in University of Yamanashi” established by the Animal experimentation facility, University of Yamanashi, and was approved by the Institutional Animal Care and Use Committee of the University of Yamanashi. Male C57BL6 mice were purchased from CLEA Japan (Tokyo, JAPAN). TRα-deficient mice (TRα^0/0^) were maintained at the University of Chicago for several generations and were back-crossed into the C57BL/6 background more than 10 times before TRα^0/0^ mice used for the experiments were obtained^41^. UUO was performed as previously described^43^. Three or 7 days following surgery, mice were sacrificed, and the obstructed and contralateral non-obstructed kidneys were harvested for analysis. Paraffin sections (4 μm thick) were stained with anti-α-smooth muscle actin antibodies (Abcam, #5694), anti-F4/80 antibodies (Serotec #MCA497G), or iNOS antibodies. Adult C57BL/6 male mice were irradiated twice with dose of 500 rads of irradiation from a ^60^Co source at a 3-hour interval. The mice were then intravenously injected with 1 × 10^6^ bone marrow cells from TRα^0/0^ or wild-type mice. Seven weeks later, the reconstituted mice were used for UUO experiments.

Hyperthyroid mice were intra-peritoneally injected with T3 (250 μg/kg/body weight) every day following UUO. Azithromycin, an NF-κB inhibitor, was purchased from Sigma and 50 mg/kg body weight or vehicle was injected intra-peritoneally into BM-TRαKO mice with or without UUO. The treatment protocol including the effective dosages was according to a previous report^18^.

### Hypothyroid mice

Mice were divided into 2 groups, each of which consisted of 4–6 C57BL/6 mice. At the beginning of the experiment, both groups of animals were given a low-iodine diet (LID) (CREA Japan) for 14 days to accustom them to the synthetic chow. Hypothyroidism was then induced in the mice via the addition of 0.05% methimazole and 1% potassium perchlorate to their drinking water for 21 days while they remained on the LID diet. This treatment lowered the serum-free T3 level to 2 pmol/l.

### Flow cytometric analysis

Murine kidneys were harvested and minced, followed by incubation with 0.1% collagenase type 1 (Gibco, 17100-017) for 40 min at 37 °C. Single-cell suspensions were obtained via filtration through a 70 μm cell strainer. The cells were subsequently incubated with mouse seroblock FcR^®^ (AbD Serotec, BUF041A) to inhibit binding to the Fc receptor, and were then stained with fluorescently labeled 7-AAD, anti-CD11b and anti-Ly6c (BD Biosciences) and subjected to fluorescent cell-sorting^44^. All flow cytometric analyses and cell sorting experiments were performed using a FACSaria II (BD Biosciences).

### Apoptotic cell assay

To detect apoptotic cells in UUO-kidneys, paraffin embedded kidneys were processed for TUNEL staining using the DeadEnd Colorimetric Apoptosis Detection Kit (Promega) according to the manufacturer’s instructions. TUNEL positive cells that exhibited at least one of the following features were considered to be apoptotic: nuclear chromatin marginalization and fragmentation; nuclear and cellular condensation and blebbing; and the presence of membrane-bound apoptotic bodies with preserved organelles and occasional nuclear fragments.

### Co-culture of activated macrophages and renal tubular cells

Bone marrow-derived macrophages isolated from obstructed kidneys were first seeded (1 × 10^6 ^cells/well) on Transwell inserts (BD Biosciences, #353502). The inserts were then placed into a Transwell companion plate seeded with 5 × 10^5 ^cells/well from a murine kidney epithelial cell line (M1 cells). After co-culture for 12 hours, the protein expression of neutrophil gelatinase-associated lipocalin (NGAL) was analyzed using Western blotting.

### Western blot analysis and real-time PCR

The protocols for Western blot analysis and real-time PCR were previously described^45^. Nuclear or cytoplasmic cell extracts were prepared using the NE-PER kit (PIERCE) according to the manufacturer’s protocol. The primary antibodies used for Western blot analysis were as follows: anti-phosphorylated IκB, total IκB, phosphorylated p38, IL-1 Receptor (IL1R), and total p38 antibodies, which were purchased from Cell Signaling Biotechnologies. Anti-p65, tubulin, histone 3, and MKP1, NGAL antibodies were purchased from Santa Cruz.

### Statistics

Data are expressed as the means ± SD. Statistical analysis was performed with one-way ANOVA or the unpaired two-tailed Student’s *t*-test. Probability values of less than 0.05 were considered statistically significant.

## Additional Information

**How to cite this article:** Furuya, F. *et al*. The ligand-bound thyroid hormone receptor in macrophages ameliorates kidney injury via inhibition of nuclear factor-κB activities. *Sci. Rep.* **7**, 43960; doi: 10.1038/srep43960 (2017).

**Publisher's note:** Springer Nature remains neutral with regard to jurisdictional claims in published maps and institutional affiliations.

## Supplementary Material

Supplementary Information
