# Supplementary material for: The ligand-bound thyroid hormone receptor in macrophages ameliorates kidney injury via inhibition of nuclear factor-κB activities
Source: Sci Rep. 2017 Mar 8;7:43960. doi: 10.1038/srep43960 (PMC5341020; doi:10.1038/srep43960)
Supplement: Supplementary Information [file srep43960-s1.pdf]

The ligand-bound thyroid hormone receptor in macrophages ameliorates kidney injury via inhibition of nuclear factor-κB activities

Fumihiko Furuya[1], Toshihisa Ishii[1], Shogo Tamura[2], Kazuya Takahashi[1], Hidetoshi Kobayashi[1], Masashi Ichijo[1], Soichi Takizawa[1], Masahiro Kaneshige[1], Katsue Suzuki-Inoue[2], and Kenichiro Kitamura[1]

1. Third Department of Internal Medicine, Interdisciplinary Graduate School of Medicine and Engineering, University of Yamanashi, 1110 Shimokato, Chuo, Yamanashi, 4093898, JAPAN

2. Department of Laboratory and Medicine, Interdisciplinary Graduate School of Medicine and Engineering, University of Yamanashi, 1110 Shimokato, Chuo, Yamanashi, 4093898, JAPAN

Address correspondence to: Fumihiko Furuya, Third Department of Internal Medicine, Interdisciplinary Graduate School of Medicine and Engineering, University of Yamanashi, 1110 Shimokato, Chuo, Yamanashi, 4093898, JAPAN

Phone: +81-55273-9602; Fax: +81-55273-9685; Email: ffuruya@yamanashi.ac.jp

Supplementary Figure 1

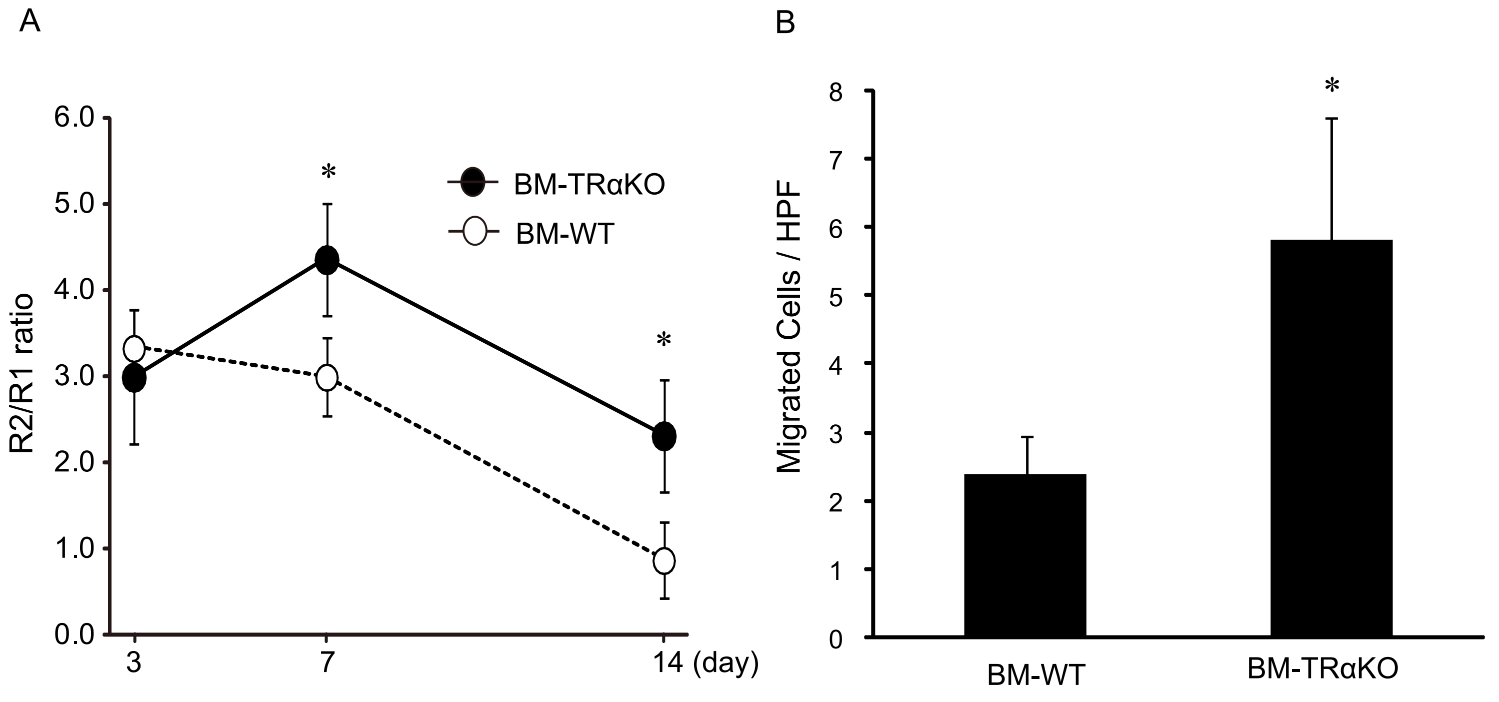

Supplementary Figure 2

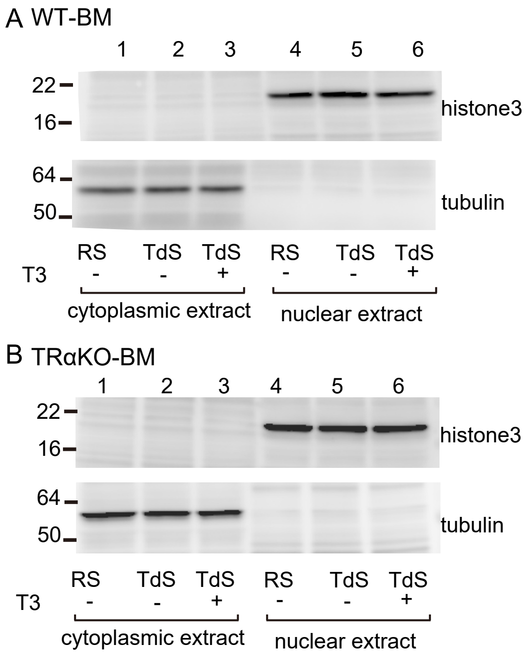

Suppl. Figure 1. A. The ratio of $CD11b^{+}Ly6C^{low}$ (R1) and $CD11b^{+}Ly6C^{high}$ (R2) cells, which are subpopulations of $CD11b^{+}Ly6C^{+}$ cells (Q2) that were isolated from the kidneys of BM-TRα KO or BM-WT mice at the indicated times after subjection of the mice to UUO, is shown. The number of cells was quantified using FACS AriaII (BioRad). B. Macrophages (5000 cells) derived from the UUO kidneys of BM-TRα KO or BM-WT mice were plated in the insert of Boyden chambers. The numbers of macrophages that migrated through the porous membranes during the 8-h incubation are indicated (n=6). All data are expressed as the means ± S.D. (error bars). *p<0.05.

Suppl. Figure 2. Bone marrow-derived macrophages of WT-BM (A) or TRα KO-BM (B) were pooled from 6 mice and incubated for 24 h in 10% serum (RS), stripped serum (TdS), or TdS with 30 nM of T3, followed by separation of the cell extract into nuclear and cytosolic fractions and western blotting. The expression of histone 3 or tubulin was used as a loading control for the cytosolic and nuclear fractions.
